# Supplementary material for: Genome-Wide Identification and Expression Analysis of the SWEET Gene Family in Capsicum annuum L
Source: Int J Mol Sci. 2023 Dec 12;24(24):17408. doi: 10.3390/ijms242417408 (PMC10744294; doi:10.3390/ijms242417408)
Supplement: Supplementary file 1 [file ijms-24-17408-s001.zip › ijms-2715667-supplementary.pdf]

Supplemental Table S1. The full-length sequence primers of *CaSWEET16/22/31*

Gene Name	Primer
CaSWEET16	F: tttggagaggacacgctcgagATGATTACATTCAAAAGGATTATCACG
	R: ataatccatgaattcctcgagTACTCCGCAATCCAGCTGACT
CaSWEET22	F: tttggagaggacacgctcgagATGGTTAACCTTAGTCACTTAGCTC
	R: ataatccatgaattcctcgagGGTTTCCACAGTTTGCAACT
CaSWEET31	F: tttggagaggacacgctcgagATGGCCATATTTGACCTCCAC
	R: ataatccatgaattcctcgagTGCAGCACACACGACAAGC

F: Forward; R: Reverse
